# Bayesian estimation of the parameter of Maxwell-Mukherjee Islam distribution using assumptions of the Extended Jeffrey's, Inverse-Rayleigh and Inverse-Nakagami priors under the three loss functions

**DOI:** 10.1016/j.heliyon.2021.e08200

**Published:** 2021-10-19

**Authors:** Aliyu Ismail Ishaq, Alfred Adewole Abiodun, Jamilu Yunusa Falgore

**Affiliations:** a Department of Statistics, Ahmadu Bello University, Zaria, Nigeria; b Department of Statistics, University of Ilorin, Ilorin, Nigeria

**Keywords:** Extended Jeffrey's prior, Inverse-Nakagami prior, Inverse-Rayleigh prior, Maxwell generalized family, Maxwell-Mukherjee Islam distribution

## Abstract

A three-parameter Maxwell-Mukherjee Islam distribution was proposed by applying Maxwell generalized family of distributions introduced by Ishaq and Abiodun [17]. The probability density and cumulative distribution functions of the proposed distribution were defined. The validity test was derived from its cumulative distribution function. The study aimed to obtain a Bayesian estimation of the scale parameter of Maxwell-Mukherjee Islam distribution by using assumptions of the Extended Jeffrey's (Uniform, Jeffrey's and Hartigan's), Inverse-Rayleigh and Inverse-Nakagami priors under the loss functions, namely, Squared Error Loss Function (SELF), Precautionary Loss Function (PLF) and Quadratic Loss Function (QLF), and their performances were compared. The posterior distribution under each prior and its corresponding loss functions was derived. The performance of the Bayesian estimation was illustrated from the basis of quantile function by using a simulation study and application to real life data set. For different sample sizes and parameter values, the QLF and SELF under Jeffrey's and Hartigan's priors produced the same estimates, bias and Mean Squared Error (MSE) just as we observed in their mathematical derivatives. Similarly, the SELF, PLF and QLF under Inverse-Rayleigh and Inverse-Nakagami priors provided the same performance when some parameter values are equal. For some parameter values, the QLF under Inverse-Nakagami and Inverse-Rayleigh priors produced the least values of MSE. In the application to real life data set, the QLF and SELF under Jeffrey's and Hartigan's priors; the SELF, PLF and QLF under Inverse-Rayleigh and Inverse-Nakagami priors provided similar results as observed in the simulation study. Therefore, the study concluded that the QLF under Inverse-Rayleigh and Inverse-Nakagami priors could effectively be used in the estimation of scale parameter of Maxwell-Mukherjee Islam distribution using Bayesian approach.

## 1 Introduction

Mukherjee and Islam [21] developed a continuous statistical distribution popularly known as Mukherjee-Islam distribution that has a finite range of values suitable for modeling datasets related to failure time, reliability analysis, and life testing among others. This distribution has decreasing and increasing failure rates. The mathematical form of this distribution can be computed easily compared to normal, gamma, and beta distributions. Mukherjee-Islam distribution is flexible and approaches many distributions like uniform and exponential models. Many researchers have considered the Mukherjee-Islam model as a lifetime distribution and applied it in various areas of statistical applications including Siddiqui [29] who studied the physical properties of the Mukherjee-Islam distribution, also discovered that the distribution could be monotonic decreasing and exhibit unimodal failure rates. The modification of Mukherjee-Islam distribution was discussed in Siddiqui et al. [28] by applying Lawless's approach, see Lawless [20]. Khan [19] obtained Reliability and Bayesian estimation of the parameter of Mukherjee-Islam distribution using assumptions of inverted gamma, uniform, and Siddiqui's priors, and the posterior distribution of each estimate were obtained in the literature. An extension of Mukherjee-Islam distribution was proposed by Al-Zou'bi [4] using quadratic transmutation map introduced by Shaw [27] and they studied Transmuted Mukherjee-Islam distribution. The density plot of this distribution has left skewed with an increasing failure rate. The order statistics, moments, entropies, moment generating function, and estimates of the parameters were derived. A three-parameter lifetime distribution was introduced by Rather [24] by adding an extra shape parameter to study Exponentiated Mukherjee-Islam distribution. Several properties of this distribution including moments, harmonic mean, moment generating function, Renyi, and Shanon entropies were discussed. The maximum likelihood estimators, Fisher information matrix, and likelihood ratio test have been studied in the literature.

According to Mukherjee [21], a random variable *X* is said to have Mukherjee-Islam distribution if its cumulative distribution function (cdf) is given by

(1) $$T(x;a,b)={\left(\frac{x}{a}\right)}^{b},\;0<x\leq a$$

and

(2) $$t(x;a,b)=\frac{b}{a^{b}}x^{{}^{b-1}},\;0<x\leq a$$

respectively, where *a* and *b* are positive scale and shape parameters respectively.

A three-parameter lifetime statistical distribution referred to as Maxwell-Mukherjee Islam (MMI) distribution is proposed in this study which serves as an extension of Mukherjee-Islam distribution by applying Maxwell generalized family of distributions developed by Ishaq and Abiodun [17].

The motivations behind this study are as follows:

i. to propose a new continuous probability distribution referred to as Maxwell-Mukherjee Islam distribution by using Maxwell generalized family of distribution introduced by Ishaq and Abiodun [17]
ii. to test the validity of the proposed Maxwell-Mukherjee Islam distribution
iii. to estimate the parameter of the new distribution by using Bayesian estimation under three loss functions, and finally
iv. to explore the most appropriate loss function among the three-loss functions that can be used effectively in the Bayesian estimation of the parameter of Maxwell Mukherjee-Islam distribution.

This study is organized as follows: In section 2, we define the cumulative distribution and probability density function of the Maxwell-Mukherjee Islam distribution by using the Maxwell generalized family of distributions. The validity test and maximum likelihood estimation of the proposed distribution were carried out. The prior and posterior distributions are defined in section 3. The Extended Jeffrey's (Uniform, Jeffrey's, and Hartigan's), Inverse-Rayleigh and Inverse-Nakagami are provided in section 4. The loss functions (Squared Error Loss Function (SELF), Precautionary Loss Function (PLF), and Quadratic Loss Function (QLF)) of each prior are presented in section 5. Section 6 provides a simulation study and the application to real life data set. The discussion of results and conclusion are given in section 7.

## 2 Maxwell generalized family of distributions

This section introduces Maxwell generalized family of distributions developed by Ishaq and Abiodun [17]. The distribution and density functions of this family of distributions are derived from the logit of the Maxwell model. A new compound model referred to as Maxwell-Weibull distribution has been proposed in the literature by using Maxwell generalized family of distributions. This distribution serves as an alternative to Marshall-Olkin extended Weibull, Weibull Rayleigh, and Weibull Exponential models in terms of application to real-life data sets.

As proposed by Ishaq and Abiodun [17], the cumulative distribution function of Maxwell generalized family of distributions is given by

(3) $$F\left(x;c,\phi \right)=\frac{2}{\sqrt{\pi }}\gamma \left(\frac{3}{2},\;\;\frac{1}{2c^{2}}{\left(\frac{T\left(x;\phi \right)}{1-T\left(x;\phi \right)}\right)}^{2}\right),\;-\infty <x<\infty$$

where *c* is a positive scale parameter, T(x;ϕ) is the baseline cdf with a vector parameter *ϕ*. The corresponding probability density function of the Maxwell generalized family of distributions is

(4) $$f\left(x;c,\phi \right)=\frac{2t\left(x;\phi \right)}{c^{3}\sqrt{2\pi }{\left(1-T\left(x;\phi \right)\right)}^{2}}{\left(\frac{T\left(x;\phi \right)}{1-T\left(x;\phi \right)}\right)}^{2}\;\;\times \exp\left(-\frac{1}{2c^{2}}{\left(\frac{T\left(x;\phi \right)}{1-T\left(x;\phi \right)}\right)}^{2}\right)$$

where t(x;ϕ) is the baseline pdf. Ishaq and Abiodun [16] considered the pdf defined in (4) and studied Maxwell-Dagum distribution.

### 2.1 The Maxwell-Mukherjee Islam distribution

The proposed Maxwell-Mukherjee Islam distribution is provided in this section by applying the cdf and pdf of the family of distributions as given respectively in (3) and (4).

#### 2.1.1 The cdf and pdf of the Maxwell-Mukherjee Islam distribution

The proposed cdf of the Maxwell-Mukherjee Islam distribution is obtained by inserting the baseline cdf defined in (1) into (3) as

(5) $$F\left(x;a,b,c\right)=\frac{2}{\sqrt{\pi }}\gamma \left(\frac{3}{2},\;\;\frac{1}{2c^{2}}{\left(\frac{{\left(\frac{x}{a}\right)}^{b}}{1-{\left(\frac{x}{a}\right)}^{b}}\right)}^{2}\right),\;0<x\leq a$$

where a,c are positive scale parameters and *b* is a positive shape parameter. The corresponding pdf is obtained by inserting the baseline cdf and pdf defined in (1) and (2) into (4) as

(6) $$f\left(x;a,b,c\right)=\frac{2bx^{{}^{b-1}}}{a^{b}c^{3}\sqrt{2\pi }{\left(1-{\left(\frac{x}{a}\right)}^{b}\right)}^{2}}{\left(\frac{{\left(\frac{x}{a}\right)}^{b}}{1-{\left(\frac{x}{a}\right)}^{b}}\right)}^{2}\;\;\times \exp\left(-\frac{1}{2c^{2}}{\left(\frac{{\left(\frac{x}{a}\right)}^{b}}{1-{\left(\frac{x}{a}\right)}^{b}}\right)}^{2}\right)$$

**Figure 1:**
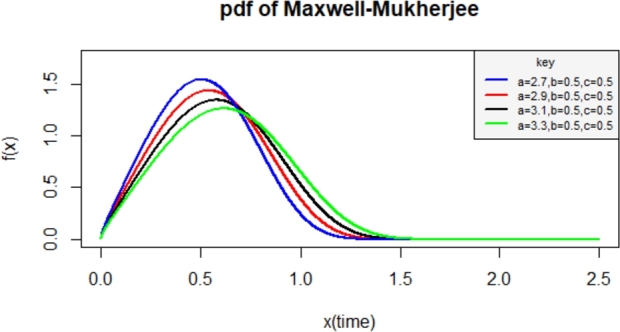
pdf of the Maxwell-Mukherjee Islam distribution for different parameter values.

**Figure 2:**
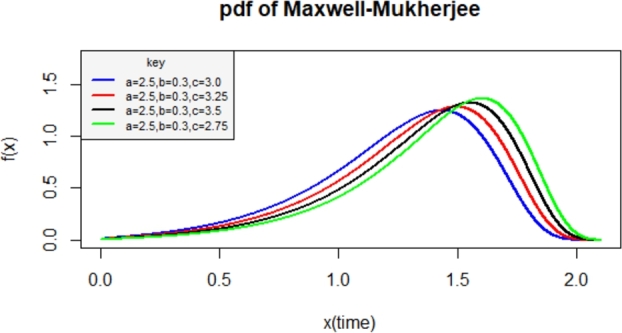
pdf of the Maxwell-Mukherjee Islam distribution for different parameter values.

We can denote the pdf in equation (6) as X∼MMI(a,b,c) which reads *X* follows Maxwell-Mukherjee Islam distribution with parameters a,b and *c*. Various plots of the pdf of Maxwell-Mukherjee Islam distribution are presented in Figure 1, Figure 2.

The Maxwell-Mukherjee Islam density plots can be left-skewed and right-skewed as observed in Figure 1, Figure 2.

#### 2.1.2 Validity test of the Maxwell-Mukherjee Islam distribution

To show that the proposed Maxwell Mukherjee-Islam distribution is a valid statistical distribution, we apply the following properties of the CDF of any continuous probability distribution as

i. $\underset{x\to \;-\infty }{\lim}\;F\left(x;a,b,c\right)=0$
ii. $\underset{x\to \;\infty }{\lim}\;F\left(x;a,b,c\right)=1$

Now, if the cdf of the proposed distribution defined in (5) satisfies all the above properties, we can say that the Maxwell-Mukherjee Islam distribution is a valid continuous statistical distribution.

Proof (7) $$\underset{x\to \;-\infty }{\lim}\;F\left(x;a,b,c\right)=\frac{2}{\sqrt{\pi }}\underset{x\to 0}{\lim}\;\gamma \left(\frac{3}{2},\;\;\frac{1}{2c^{2}}{\left(\frac{{\left(\frac{x}{a}\right)}^{b}}{1-{\left(\frac{x}{a}\right)}^{b}}\right)}^{2}\right)=\frac{2}{\sqrt{\pi }}\underset{x\to 0}{\lim}\;\;\;\gamma \left(k,\;\;v\right)$$

where $k=\frac{3}{2}$ and $v=\frac{1}{2c^{2}}{\left(\frac{{\left(\frac{x}{a}\right)}^{b}}{1-{\left(\frac{x}{a}\right)}^{b}}\right)}^{2}$. This implies,

(8) $$\underset{x\to 0}{\lim}\;v=\frac{1}{2c^{2}}\underset{x\to 0}{\lim}\;{\left(\frac{{\left(\frac{x}{a}\right)}^{b}}{1-{\left(\frac{x}{a}\right)}^{b}}\right)}^{2}=\frac{1}{2c^{2}}\times 0=0$$

Therefore, (7) can be expressed as

(9) $$\underset{x\to \;-\infty }{\lim}\;F\left(x;a,b,c\right)=\frac{2}{\sqrt{\pi }}\;\;\gamma \left(k,\;\;0\right)=0$$

which completes the proof of the property (i), referred to Gradshteyn and Ryzhik [14] section 8.350 for the details of incomplete gamma function given in (9). For the second property, the cdf in (5) can be simplified as

(10) $$\underset{x\to \;\;\infty }{\lim}\;F\left(x;a,b,c\right)=\frac{2}{\sqrt{\pi }}\underset{x\to a}{\lim}\;\;\;\gamma \left(k,\;\;v\right).$$

Therefore, the upper limit of *v* is

(11) $$\underset{x\to a}{\lim}\;v=\frac{1}{2c^{2}}\underset{x\to a}{\lim}\;{\left(\frac{{\left(\frac{x}{a}\right)}^{b}}{1-{\left(\frac{x}{a}\right)}^{b}}\right)}^{2}=\frac{1}{2c^{2}}\times \infty =\infty$$

hence, (10) can be presented as

(12) $$\underset{x\to \;\infty }{\lim}\;F\left(x;a,b,c\right)=\frac{2}{\sqrt{\pi }}\;\;\gamma \left(k,\;\infty \right)$$

The incomplete gamma function in (12) has been defined in Gradshteyn and Ryzhik [14] section 8.356. Therefore, (12) can be written as

(13) $$\underset{x\to \;\infty }{\lim}\;F\left(x;a,b,c\right)=\frac{2}{\sqrt{\pi }}\left\{\Gamma \left(k\right)-\Gamma \left(k,\;\infty \right)\right\}=\frac{2}{\sqrt{\pi }}\times \Gamma \left(k\right)=\frac{2}{\sqrt{\pi }}\;\;\times \Gamma \left(\frac{3}{2}\right)\;\;=\;\;\frac{2}{\sqrt{\pi }}\;\;\times \frac{1}{2}\times \Gamma \left(\frac{1}{2}\right)\;=1,$$

which completes the proof of the property (ii). This implies that the Maxwell Mukherjee - Islam distribution is a valid continuous probability distribution.

### 2.2 Maximum likelihood estimation

The estimate of the scale parameter *c* of Maxwell-Mukherjee Islam distribution can be obtained by using the Maximum likelihood method.

Let $X_{1},X_{2},.....,X_{n}$ be a random sample of size n drawn from Maxwell-Mukherjee Islam distribution with parameters given in vector form as $\zeta ={\left(a,b,c\right)}^{T}$. The likelihood function of the Maxwell-Mukherjee Islam distribution is the joint density of the random variables $X_{i},\;i=1,2,...,n$ as

(14) $$L\left(x|a,b,c\right)={\left(\frac{2b}{a^{b}c^{3}\sqrt{2\pi }}\right)}^{n}\prod_{i=1}^{n}\left[\frac{x_{i}^{{}^{b-1}}}{{\left(1-{\left(\frac{x_{i}}{a}\right)}^{b}\right)}^{2}}{\left(\frac{{\left(\frac{x_{i}}{a}\right)}^{b}}{1-{\left(\frac{x_{i}}{a}\right)}^{b}}\right)}^{2}\right]\;\times \;\prod_{i=1}^{n}\exp\left(-\frac{1}{2c^{2}}\sum_{i=1}^{n}{\left(\frac{{\left(\frac{x_{i}}{a}\right)}^{b}}{1-{\left(\frac{x_{i}}{a}\right)}^{b}}\right)}^{2}\right)$$

The log-likelihood function of (14), that is log⁡L(x|a,b,c)=ℓ(x|a,b,c) is given by

(15) $$\ell \left(x|a,b,c\right)\;=n\log\left(2\right)+n\log\left(b\right)-nb\log\left(a\right)-3n\log\left(c\right)-\frac{n}{2}\log\left(2\pi \right)+\left(b-1\right)\;\times \;\sum_{i=1}^{n}\log\left(x_{i}\right)-2\sum_{i=1}^{n}\left(\log\left(1-\varphi_{i}\right)\right)+\sum_{i=1}^{n}\left(\log\left(z_{i}^{2}\right)\right)-\frac{1}{2c^{2}}\sum_{i=1}^{n}z_{i}^{2}$$

where $\varphi_{i}={\left(\frac{x_{i}}{a}\right)}^{b}$ and $z_{i}=\frac{\varphi_{i}}{1-\varphi_{i}}$.

Similarly, the likelihood function for scale parameter *c* of the Maxwell-Mukherjee Islam distribution is given by

(16) $$L\left(x|c\right)=Kc^{-3n}\exp\left(-\frac{1}{2c^{2}}\sum_{i=1}^{n}z_{i}^{2}\right)$$

where $K={\left(\frac{2b}{a^{b}\sqrt{2\pi }}\right)}^{n}\prod_{i=1}^{n}\left[\frac{x_{i}^{{}^{b-1}}\varphi_{i}^{2b}}{{\left(1-\varphi_{i}\right)}^{4}}\right]$. The log-likelihood function of (16), that is log L(x|c)=ℓ(x|c) is given by

(17) $$\ell \left(x|c\right)=n\log\left(K\right)-3n\log\left(c\right)-\frac{1}{2c^{2}}\sum_{i=1}^{n}z_{i}^{2}$$

Therefore, the maximum likelihood estimator of the scale parameter *c* can be obtained by differentiating (17) partially with respect to parameter *c* and setting the result to zero as

(18) $$\frac{\partial \ell }{\partial c}=\frac{-3n}{c}+\frac{1}{c^{3}}\sum_{i=1}^{n}z_{i}^{2}=0$$

This implies

(19) $$-3nc^{2}+\sum_{i=1}^{n}z_{i}^{2}=0$$

Thus, the maximum likelihood estimator of the scale parameter *c* is obtained from (19) as

(20) $$\overset{ˆ}{c}=\sqrt{\frac{1}{3n}\sum_{i=1}^{n}z_{i}^{2}}=\sqrt{\frac{1}{3n}\sum_{i=1}^{n}{\left(\frac{{\left(\frac{x_{i}}{a}\right)}^{b}}{1-{\left(\frac{x_{i}}{a}\right)}^{b}}\right)}^{2}}$$

which is the estimate of the scale parameter *c* of Maxwell-Mukherjee Islam distribution expressed in terms of *a* and *b*.

## 3 Bayesian estimation of the scale parameter *c* of Maxwell-Mukherjee Islam distribution

In this section, the Bayesian Estimation of the scale parameter *c* of Maxwell-Mukherjee Islam distribution is provided by using the Bayesian estimation method.

### 3.1 Bayesian estimation method

The Bayesian estimation method has received a vital role application for analyzing failure time data in statistical inference that has been proposed in the past as an alternative to the traditional methods of estimation. The Bayesian estimation requires knowledge about prior (s) distribution for the parameter (s) and the availability of the dataset.

### 3.2 Prior and posterior distribution

#### 3.2.1 Prior distributions

Let $X_{1},X_{2},.....,X_{n}$ be a random sample of size n drawn from a probability distribution with density function given by g(x|ϖ), where *ϖ* is unknown parameter which needs to be estimated. The prior distribution denoted g(ϖ) captures information about the parameter *ϖ*.

#### 3.2.2 Posterior distribution

By applying Bayes' Theorem, the posterior distribution of the parameter *ϖ* is the conditional density given by

(21) $$\rho \left(\varpi |x\right)=\frac{g(\varpi )L\left(x|\varpi \right)}{\int_{-\infty }^{\infty }g(\varpi )L\left(x|\varpi \right)d\varpi }$$

where L(x|ϖ) and g(ϖ) are likelihood function and prior distribution respectively.

A number of researches have been carried out in distributional statistics using both Bayesian and classical techniques. For instance, Feroze and Aslam [13] considered the Error function as a lifetime distribution and obtained its scale parameter by using informative and non-informative priors. The Bayes estimators and their associated Risk factors of the Error function have been derived by using different priors including Chi-square, Normal, Maxwell, Uniform, Rayleigh and Jeffrey's priors. The performance of the Bayes estimators was illustrated by using a simulation study. It was discovered that for the point estimation, the use of the Maxwell prior under entropy loss function produced the best estimates irrespective of sample sizes, while for interval estimation the chi-square prior can be employed. The effects of underestimation and overestimation of the shape parameter of Pareto distribution was studied by Saxena [26] under assumptions of exponential, erlang and doubly truncated gamma priors using different loss functions. The posterior distribution and its corresponding Bayes estimators of the different priors were derived. The performance of the Bayes estimators were studied by using real life data set. It was concluded that some asymmetric LINEX loss functions provided great efficiency than others. The Bayes estimators of the parameters of Rayleigh distribution was obtained by Dey and Maiti [11] under symmetric and asymmetric loss functions. The Highest Posterior Density (HPD), Bayes predictive estimation and HPD prediction interval were studied. The Bayes estimator under Jeffrey's prior provides overestimation when the sample size is small whereas a Bayes estimator under Hartigan's prior produced underestimation for a large sample size. Dar et al. [8] studied a Bayesian estimation of the parameter of Maxwell distribution using assumptions of Gamma and Extended Jeffrey's priors under stein's, Al-Bayyat's, squared error and precautionary loss functions. A simulation study was conducted for different sample sizes and parameter values. It was found that the Bayes estimators under stein's loss function produced the best estimates by having minimum mean squared errors. Similarly, Al-Bayyat's loss function proved to be better estimated in comparison with other loss functions.

Several other authors who have worked on Bayesian methods of estimation include Dey [9] who obtained the Bayesian estimation and the associated Risk factors of the shape parameter of the generalized exponential distribution. Danish [7] used the Gibbs sampling scheme technique to obtain the parameters of the Weibull model. Zaka and Akhter [30] discussed Bayesian estimation of the parameter of the Power function model under assumptions of Pareto, chi-square, quasi, Jeffrey's and exponential priors. The parameter of the Exponential distribution was studied by Hasan and Baizid [15] by using classical and non-classical methods of estimation. Kamaljit and Kalpana [18] applied Bayesian and Semi-Bayesian approaches to estimate the parameters of the Generalized Inverse Weibull distribution. Other researches can be found in Dey and Maiti [10], Rasheed [23], AlBaldawi [5], Adegoke et al. [1], Eraikhuemen et al. [12], Aijaz et al. [2] and many more.

This study considers the Extended Jeffrey's (Uniform, Jeffrey's and Hartigan's), Inverse-Rayleigh and Inverse-Nakagami priors as the prior distributions.

## 4 The Extended Jeffrey's, Inverse-Rayleigh and Inverse-Nakagami prior distributions

### 4.1 Extended Jeffrey's prior

As proposed by Al-Kutubi [3], the extended Jeffrey's prior is defined as

(22) $$g(\varpi )\propto {\left[I\left(\varpi \right)\right]}^{h},\;\;\;\;\;\;\;\;\;h\geq 0$$

where $I\left(\varpi \right)=-E\left(\frac{\partial^{2}\ell }{\partial \varpi^{2}}\right)$ is the Fisher Information Matrix. The Uniform, Jeffrey's, and Hartigan's priors can be obtained respectively by setting h=1,1/2 and 3/2. To determine the Fisher Information Matrix, we differentiate (18) partially with respect to parameter *c* as

(23) $$\frac{\partial^{2}\ell }{\partial c^{2}}=\frac{3n}{c^{2}}-\frac{3}{c^{4}}\sum_{i=1}^{n}z_{i}^{2}$$

The expected term of (23) is determined as

(24) $$E\left(\frac{\partial^{2}\ell }{\partial c^{2}}\right)=\int_{-\infty }^{\infty }\left(\frac{3n}{c^{2}}-\frac{3}{c^{4}}\sum_{i=1}^{n}z_{i}^{2}\right)f(x)dx=\frac{3n}{c^{2}}\int_{-\infty }^{\infty }f(x)dx-\frac{3n}{c^{4}}\int_{-\infty }^{\infty }z^{2}f(x)dx$$

The integral $\int_{-\infty }^{\infty }f(x)dx$ in (24) can be expressed as

(25) $$\int_{-\infty }^{\infty }f\left(x\right)dx=\frac{2b}{a^{b}c^{3}\sqrt{2\pi }}\int_{0}^{a}\frac{x^{{}^{b-1}}}{{\left(1-{\left(\frac{x}{a}\right)}^{b}\right)}^{2}}{\left(\frac{{\left(\frac{x}{a}\right)}^{b}}{1-{\left(\frac{x}{a}\right)}^{b}}\right)}^{2}\;\;\times \exp\left(-\frac{1}{2c^{2}}{\left(\frac{{\left(\frac{x}{a}\right)}^{b}}{1-{\left(\frac{x}{a}\right)}^{b}}\right)}^{2}\right)dx$$

Let

(26) $$\rho ={\left(\frac{x}{a}\right)}^{b},\;\;\;\;\Rightarrow \;\;\;dx=\frac{a^{b}}{bx^{b-1}}d\rho$$

then inserting (26) into (25) gives

(27) $$\int_{-\infty }^{\infty }f\left(x\right)dx=\frac{2}{c^{3}\sqrt{2\pi }}\int_{0}^{1}\frac{1}{{\left(1-\rho \right)}^{2}}{\left(\frac{\rho }{1-\rho }\right)}^{2}\exp\left(-\frac{1}{2c^{2}}{\left(\frac{\rho }{1-\rho }\right)}^{2}\right)d\rho$$

Also, let

(28) $$y=\frac{1}{2c^{2}}{\left(\frac{\rho }{1-\rho }\right)}^{2},\;\;\;\;\Rightarrow \;\;\;d\rho =\frac{c^{2}{\left(1-\rho \right)}^{3}}{\rho }dy,$$

Substituting (28) into (27) becomes

(29) $$\int_{-\infty }^{\infty }f\left(x\right)dx=\frac{2}{c\sqrt{2\pi }}\int_{0}^{\infty }\left(\frac{\rho }{1-\rho }\right)e^{-y}dy=\frac{2}{c\sqrt{2\pi }}\int_{0}^{\infty }{\left(2c^{2}y\right)}^{\frac{1}{2}}e^{-y}dy=\frac{2}{\sqrt{\pi }}\int_{0}^{\infty }y^{\left(\frac{1}{2}+1\right)-1}e^{-y}dy=1$$

Therefore, equation (24) can be simplified as

(30) $$E\left(\frac{\partial^{2}\ell }{\partial c^{2}}\right)=\frac{3n}{c^{2}}\times 1-\frac{6nb}{a^{b}c^{7}\sqrt{2\pi }}\int_{0}^{a}\frac{x^{b-1}}{{\left(1-{\left(\frac{x}{a}\right)}^{b}\right)}^{2}}{\left(\frac{{\left(\frac{x}{a}\right)}^{b}}{1-{\left(\frac{x}{a}\right)}^{b}}\right)}^{4}\;\;\times \;\exp\left(-\frac{1}{2c^{2}}{\left(\frac{{\left(\frac{x}{a}\right)}^{b}}{1-{\left(\frac{x}{a}\right)}^{b}}\right)}^{2}\right)dx$$

Let

(31) $$w=\frac{{\left(\frac{x}{a}\right)}^{b}}{1-{\left(\frac{x}{a}\right)}^{b}},\;\;\;\Rightarrow \;\;\;\;dx=\frac{a^{b}{\left(1-{\left(\frac{x}{a}\right)}^{b}\right)}^{2}}{bx^{b-1}}dw$$

By inserting (31) into (30) gives

(32) $$E\left(\frac{\partial^{2}\ell }{\partial c^{2}}\right)=\frac{3n}{c^{2}}-\frac{6n}{c^{7}\sqrt{2\pi }}\int_{0}^{\infty }w^{4}e^{-\frac{w^{2}}{2c^{2}}}dw$$

Also, let

(33) $$v=\frac{w^{2}}{2c^{2}},\;\;\;\Rightarrow \;\;\;dw=\frac{c^{2}}{w}dv$$

Substituting (33) into (32) yields

(34) $$E\left(\frac{\partial^{2}\ell }{\partial c^{2}}\right)=\frac{3n}{c^{2}}-\frac{6n}{c^{5}\sqrt{2\pi }}\int_{0}^{\infty }w^{3}e^{-m}dv$$

(35) $$=\frac{3n}{c^{2}}-\frac{6n}{c^{5}\sqrt{2\pi }}\int_{0}^{\infty }{\left(2c^{2}v\right)}^{\frac{3}{2}}e^{-v}dv=\frac{3n}{c^{2}}-\frac{12n}{c^{2}\sqrt{\pi }}\Gamma \left(\frac{3}{2}+1\right)=\frac{-6n}{c^{2}}$$

Hence, the Fisher Information Matrix is obtained as

(36) $$I\left(c\right)=\frac{6n}{c^{2}}$$

Therefore, the Extended Jeffrey's prior is derived from (22) for ϖ=c by inserting (36) as

(37) $$g(c)\propto {\left[\frac{1}{c^{2}}\right]}^{h},\;\;\;\;\;\;\;\;\;h\geq 0$$

Thus, the Uniform, Jeffrey's, and Hartigan's priors of the scale parameter *c* of Maxwell-Mukherjee Islam distribution can be obtained from (37) as follows:

#### 4.1.1 Uniform prior

The Uniform prior is derived by setting h=0 as

(38) $$g_{1}(c)=1$$

#### 4.1.2 Jeffrey's prior

Setting h=1/2, the Jeffrey's prior is

(39) $$g_{2}(c)=\frac{1}{c}$$

#### 4.1.3 Hartigan's prior

For h=3/2, Hartigan's prior is obtained as

(40) $$g_{3}(c)=\frac{1}{c^{3}}$$

**Figure 3:**
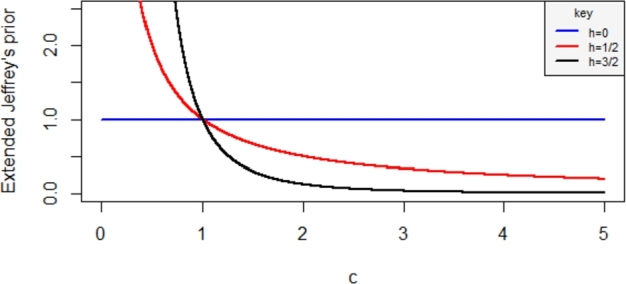
plot of the Extended Jeffrey's prior for different value of *h*.

The plot of the Extended Jeffrey's prior is presented in Fig. 3 by setting h=0,1/2 or h=3/2.

As observed in Fig. 3, the Uniform prior (h=0) has a constant shape and decreasing for Jeffrey's and Hartigan's priors when h=1/2 and h=3/2.

### 4.2 Posterior distribution of the Extended Jeffrey's prior of the estimate of scale parameter *c* of Maxwell-Mukherjee Islam distribution

To derive the posterior distribution of the Extended Jeffrey's prior, the numerator of (21) is expressed in (41) by substituting (16) and (37) for ϖ=c as

(41) $$g(c)L\left(x|c\right)=Kc^{-2h-3n}e^{-\frac{1}{2c^{2}}\sum_{i=1}^{n}z_{i}^{2}}=Kc^{-2h-3n}e^{-\frac{\vartheta }{2c^{2}}}$$

where $\vartheta =\sum_{i=1}^{n}z_{i}^{2}$. The integral of (41) is expressed as

(42) $$\int_{-\infty }^{\infty }g(c)L\left(x﹨c\right)dc=K\int_{0}^{\infty }c^{-2h-3n}e^{-\frac{\vartheta }{2c^{2}}}dc$$

Let

(43) $$\omega =\frac{\vartheta }{2c^{2}},\;\;\;\Rightarrow \;\;\;\;dc=-\frac{c^{3}}{\vartheta }d\omega$$

Inserting (43) into (42) becomes

(44) $$\int_{-\infty }^{\infty }g(c)L\left(x﹨c\right)dc=\frac{K}{\vartheta }\int_{0}^{\infty }{\left({\left(\frac{\vartheta }{2\omega }\right)}^{\frac{1}{2}}\right)}^{3-2h-3n}e^{-\omega }d\omega$$

(45) $$=\frac{2^{\frac{2h+3n-3}{2}}}{\vartheta^{\frac{2h+3n-3}{2}+1}}K\int_{0}^{\infty }\omega^{\frac{2h+3n-1}{2}-1}e^{-\omega }d\omega =\frac{2^{\frac{2h+3n-1}{2}-1}}{\vartheta^{\frac{2h+3n-1}{2}}}K\Gamma \left(\frac{2h+3n-1}{2}\right)$$

Therefore, the posterior distribution of the Extended Jeffrey's prior of the scale parameter of Maxwell-Mukherjee Islam distribution is obtained by inserting (41) and (45) into (21) as

(46) $$\rho \left(c|x\right)=\frac{c^{-2h-3n}\vartheta^{\frac{2h+3n-1}{2}}e^{-\frac{\vartheta }{2c^{2}}}}{2^{\frac{2h+3n-1}{2}-1}\Gamma \left(\frac{2h+3n-1}{2}\right)}=\frac{c^{-2h-3n}\vartheta^{m}e^{-\frac{\vartheta }{2c^{2}}}}{2^{m-1}\Gamma \left(m\right)}$$

where $m=\frac{2h+3n-1}{2}$. Therefore, the posterior distribution of the Uniform, Jeffrey's, and Hartigan's priors of the scale parameter *c* of the Maxwell-Mukherjee Islam distribution are derived by setting h=0,1/2 or 3/2 into (46) given as follows:

#### 4.2.1 Posterior distribution of the Uniform prior

The posterior distribution of the Uniform prior is derived by setting h=0 as

(47) $$\rho_{1}\left(c|x\right)=\frac{c^{-3n}\vartheta^{m}e^{-\frac{\vartheta }{2c^{2}}}}{2^{m-1}\Gamma \left(m\right)},\;\;\;\;\;\;\;\;m=\frac{3n-1}{2}$$

#### 4.2.2 Posterior distribution of the Jeffrey's prior

Setting h=1/2, the posterior distribution of the Jeffrey's prior is

(48) $$\rho_{2}\left(c|x\right)=\frac{c^{-1-3n}\vartheta^{m}e^{-\frac{\vartheta }{2c^{2}}}}{2^{m-1}\Gamma \left(m\right)},\;\;\;\;\;\;\;\;m=\frac{3n}{2}$$

#### 4.2.3 Posterior distribution of the Hartigan's prior

For h=3/2, the posterior distribution of the Hartigan's prior is

(49) $$\rho_{3}\left(c|x\right)=\frac{c^{-3-3n}\vartheta^{m}e^{-\frac{\vartheta }{2c^{2}}}}{2^{m-1}\Gamma \left(m\right)},\;\;\;\;\;\;\;\;m=\frac{3n+2}{2}$$

### 4.3 Inverse-Rayleigh prior

Rayleigh [25] developed one parameter Rayleigh distribution as applications in the physics to model wind speed data. This distribution has some relations with other distributions like Chi-square, Weibull, and many more. Suppose that *X* follows a Rayleigh distribution with parameter *λ*, then $Y=\frac{1}{X}$ has an Inverse-Rayleigh (I-R) distribution. This research considers I-R distribution as a prior distribution and can be called as I-R prior with the density function defined as

(50) $$g(c)=\frac{2\lambda }{c^{3}}\;e^{-\frac{\lambda }{c^{2}}},\;\;\;\;\;\;\;\;\;\lambda >0,\;\;\;\;\;\;\;\;\;c>0$$

### 4.4 Posterior distribution of the Inverse-Rayleigh prior of the estimate of scale parameter *c* of Maxwell-Mukherjee Islam distribution

To obtain the posterior distribution of the I-R prior, the numerator of (21) can be expressed by substituting (16) and (50) as

(51) $$g(c)L\left(x|c\right)=2\lambda Kc^{-3n-3}\;e^{-\frac{1}{c^{2}}\left(\lambda +\frac{\vartheta }{2}\right)}$$

The denominator of (21) can be expressed as

(52) $$\int_{-\infty }^{\infty }g(c)L\left(x﹨c\right)dc=2\lambda K\int_{0}^{\infty }c^{-3n-3}\;e^{-\frac{1}{c^{2}}\left(\lambda +\frac{\vartheta }{2}\right)}dc$$

Let

(53) $$y=\frac{1}{c^{2}}\left(\lambda +\frac{\vartheta }{2}\right),\;\;\;\;\;\;\;\;c={\left(y^{-1}\left(\lambda +\frac{\vartheta }{2}\right)\right)}^{\frac{1}{2}}\;\;\;\;\;\;\;\;\Rightarrow \;\;\;\;\;\;\;\;dc=\frac{-c^{3}}{2\left(\lambda +\frac{\vartheta }{2}\right)}dy$$

Inserting (53) into (52) becomes

(54) $$\int_{-\infty }^{\infty }g(c)L\left(x﹨c\right)dc=\frac{\lambda K}{\left(\lambda +\frac{\vartheta }{2}\right)}\int_{0}^{\infty }{\left({\left(y^{-1}\left(\lambda +\frac{\vartheta }{2}\right)\right)}^{\frac{1}{2}}\right)}^{-3n}e^{-y}dy=\frac{\lambda K}{{\left(\lambda +\frac{\vartheta }{2}\right)}^{1+\frac{3n}{2}}}\int_{0}^{\infty }y^{\frac{3n}{2}}e^{-y}dy=\frac{\lambda K\Gamma \left(1+\frac{3n}{2}\right)}{{\left(\lambda +\frac{\vartheta }{2}\right)}^{1+\frac{3n}{2}}}$$

Therefore, the posterior distribution of the I-R prior of the scale parameter of Maxwell-Mukherjee Islam distribution is obtained by inserting (51) and (54) into (21) as

(55) $$\rho \left(c|x\right)=\frac{2c^{-3n-3}q^{f}e^{-\frac{q}{c^{2}}}}{\Gamma \left(f\right)}$$

where $f=1+\frac{3n}{2}$ and $q=\lambda +\frac{\vartheta }{2}$.

### 4.5 Inverse-Nakagami prior

Nakagami distribution was studied by Nakagami [22] as an alternative to Gaussian and Rayleigh distributions, and can be used in the applications of telecommunication engineering, mobile radio and many more. This distribution has some similarities and differences with the gamma and Chi-square distributions. Suppose *X* follows Nakagami distribution, then $Y=\frac{1}{X}$ has Inverse-Nakagami (I-N) distribution. In this research, the I-N distribution is considered as a prior distribution and can be referred to as I-N prior with the density function given by

(56) $$g(c)=\frac{2\alpha^{\alpha }}{\beta^{\alpha }c^{2\alpha +1}\Gamma \left(\alpha \right)}\;e^{-\frac{\alpha }{\beta c^{2}}},\;\;\;\;\;\;\;\;\;\alpha >0,\;\;\;\;\;\;\;\;\;\beta >0,\;\;\;\;\;\;\;\;\;c>0$$

### 4.6 Posterior distribution of the Inverse-Nakagami prior of the estimate of scale parameter *c* of Maxwell-Mukherjee Islam distribution

To obtain the posterior distribution of the I-N prior, the numerator of (21) can be expressed by substituting (16) and (56) as

(57) $$g(c)L\left(x|c\right)=\frac{2K\alpha^{\alpha }c^{-2\alpha -3n-1}}{\beta^{\alpha }\Gamma \left(\alpha \right)}\;e^{-\frac{1}{c^{2}}\left(\frac{\alpha }{\beta }+\frac{\vartheta }{2}\right)}$$

The denominator of (21) can be expressed as

(58) $$\int_{-\infty }^{\infty }g(c)L\left(x﹨c\right)dc=\frac{2K\alpha^{\alpha }}{\beta^{\alpha }\Gamma \left(\alpha \right)}\int_{0}^{\infty }c^{-2\alpha -3n-1}\;e^{-\frac{1}{c^{2}}\left(\frac{\alpha }{\beta }+\frac{\vartheta }{2}\right)}dc$$

Let

(59) $$\nu =\frac{1}{c^{2}}\left(\frac{\alpha }{\beta }+\frac{\vartheta }{2}\right),\;\;\;\;\;\;\;\;c={\left(\nu^{-1}\left(\frac{\alpha }{\beta }+\frac{\vartheta }{2}\right)\right)}^{\frac{1}{2}}\;\;\;\;\;\;\;\;\Rightarrow \;\;\;\;\;\;\;\;dc=\frac{-c^{3}}{2\left(\frac{\alpha }{\beta }+\frac{\vartheta }{2}\right)}d\nu$$

Inserting (59) into (58) becomes

(60) $$\int_{-\infty }^{\infty }g(c)L\left(x﹨c\right)dc=\frac{K\alpha^{\alpha }}{\beta^{\alpha }\Gamma \left(\alpha \right)}\int_{0}^{\infty }{\left({\left(\nu^{-1}\left(\frac{\alpha }{\beta }+\frac{\vartheta }{2}\right)\right)}^{\frac{1}{2}}\right)}^{2-3n-2\alpha }e^{-\nu }d\nu =\frac{K\alpha^{\alpha }}{\beta^{\alpha }\Gamma \left(\alpha \right){\left(\frac{\alpha }{\beta }+\frac{\vartheta }{2}\right)}^{\frac{2\alpha +3n}{2}}}\int_{0}^{\infty }\nu^{\frac{2\alpha +3n-2}{2}}e^{-\nu }d\nu =\frac{K\alpha^{\alpha }\Gamma \left(\frac{2\alpha +3n}{2}\right)}{\beta^{\alpha }\Gamma \left(\alpha \right){\left(\frac{\alpha }{\beta }+\frac{\vartheta }{2}\right)}^{\frac{2\alpha +3n}{2}}}$$

Therefore, the posterior distribution of the Inverse-Nakagami prior of the scale parameter of Maxwell-Mukherjee Islam distribution is obtained by inserting (57) and (60) into (21) as

(61) $$\rho \left(c|x\right)=\frac{2c^{-2\alpha -3n-1}t^{j}e^{-\frac{t}{c^{2}}}}{\Gamma \left(j\right)}$$

where $j=\frac{2\alpha +3n}{2}$ and $t=\frac{\alpha }{\beta }+\frac{\vartheta }{2}$.

## 5 Loss function

Let $X_{1},X_{2},.....,X_{n}$ be a random sample of size n drawn from a probability distribution with pdf g(x|ϖ), where *ϖ* is the unknown parameter to be estimated. The loss function denoted $L\left(\overset{ˆ}{\varpi },\varpi \right)$ is the function that represents the loss, where $\overset{ˆ}{\varpi }$ is an unbiased estimator of the unknown parameter *ϖ*. There are different forms of loss functions, but the most popular ones are the squared error loss function, precautionary loss function and quadratic loss function.

### 5.1 Squared Error Loss Function (SELF)

According to Azam and Ahmad [31], the squared error loss function is defined as

(62) $$L\left(\varpi ,{\overset{ˆ}{\varpi }}_{SELF}\right)={\left(\varpi -{\overset{ˆ}{\varpi }}_{SELF}\right)}^{2}$$

The Bayes estimator of the parameter relating to (62) is given by

(63) $${\overset{ˆ}{\varpi }}_{SELF}=E\left(\varpi |x\right)=\int_{0}^{\infty }\varpi \rho \left(\varpi |x\right)d\varpi$$

where ρ(ϖ|x) is the posterior distribution defined in (21).

### 5.2 Precautionary Loss Function (PLF)

By Azam and Ahmad [31], the precautionary loss function is given by

(64) $$L\left(\varpi ,{\overset{ˆ}{\varpi }}_{PLF}\right)=\frac{{\left(\varpi -{\overset{ˆ}{\varpi }}_{PLF}\right)}^{2}}{{\overset{ˆ}{\varpi }}_{PLF}}$$

and the Bayes estimator of the parameter *ϖ* relating to (64) is

(65) $${\overset{ˆ}{\varpi }}_{PLF}={\left\{E\left(\varpi^{2}|x\right)\right\}}^{\frac{1}{2}}={\left\{\int_{0}^{\infty }\varpi^{2}\rho \left(\varpi |x\right)d\varpi \right\}}^{\frac{1}{2}}$$

### 5.3 Quadratic Loss Function (QLF)

The quadratic loss function given in Azam and Ahmad [6] is

(66) $$L\left(\varpi ,{\overset{ˆ}{\varpi }}_{QLF}\right)={\left(\frac{\varpi -{\overset{ˆ}{\varpi }}_{QLF}}{{\overset{ˆ}{\varpi }}_{QLF}}\right)}^{2}$$

The Bayes estimator relating to (66) is given by

(67) $${\overset{ˆ}{\varpi }}_{QLF}=E\left(\varpi^{-1}|x\right)/E\left(\varpi^{-2}|x\right)$$

where $E\left(\varpi^{-i}|x\right)=\int_{0}^{\infty }\varpi^{-i}\rho \left(\varpi |x\right)d\varpi$, for i=1,2.

### 5.4 Bayesian estimation of the scale parameter *c* of Maxwell-Mukherjee Islam distribution using assumptions of the Extended Jeffrey's prior under the three loss functions

#### 5.4.1 Estimation using Squared Error Loss Function (SELF)

By substituting posterior distribution defined in (46) into (63) for ϖ=c we get

(68) $${\overset{ˆ}{c}}_{SELF}=\frac{\vartheta^{m}}{2^{m-1}\Gamma \left(m\right)}\int_{0}^{\infty }c^{1-2h-3n}e^{-\frac{\vartheta }{2c^{2}}}dc$$

by inserting (43) into (68) gives

(69) $${\overset{ˆ}{c}}_{SELF}=\frac{\vartheta^{m-1}}{2^{m-1}\Gamma \left(m\right)}\int_{0}^{\infty }{\left({\left(\frac{\vartheta }{2\omega }\right)}^{\frac{1}{2}}\right)}^{4-2h-3n}e^{-\omega }d\omega =\frac{\vartheta^{m-1+\frac{4-2h-3n}{2}}}{2^{m-1+\frac{4-2h-3n}{2}}\Gamma \left(m\right)}\int_{0}^{\infty }\omega^{\frac{2h+3n-4}{2}}e^{-\omega }d\omega =\frac{\vartheta^{\frac{1}{2}}}{2^{\frac{1}{2}}\Gamma \left(m\right)}\Gamma \left(m-\frac{1}{2}\right)$$

where $m=\frac{2h+3n-1}{2}$.

#### 5.4.2 Estimation using Precautionary Loss Function (PLF)

The integral part of (65) is obtained in (70) by substituting posterior distribution defined in (46) as

(70) $$\int_{0}^{\infty }c^{2}\rho_{i}\left(c﹨X\right)dc=\frac{\vartheta^{m}}{2^{m-1}\Gamma \left(m\right)}\int_{0}^{\infty }c^{2-2h-3n}e^{-\frac{\vartheta }{2c^{2}}}dc$$

Using (43), equation (70) becomes

(71) $$\int_{0}^{\infty }c^{2}\rho_{i}\left(c﹨X\right)dc=\frac{\vartheta^{m-1}}{2^{m-1}\Gamma \left(m\right)}\int_{0}^{\infty }{\left({\left(\frac{\vartheta }{2\omega }\right)}^{\frac{1}{2}}\right)}^{5-2h-3n}e^{-\omega }d\omega =\frac{\vartheta^{m-1+\frac{5-2h-3n}{2}}}{2^{m-1+\frac{5-2h-3n}{2}}\Gamma \left(m\right)}\int_{0}^{\infty }\omega^{\frac{2h+3n-5}{2}}e^{-\omega }d\omega =\frac{\vartheta }{2\Gamma \left(m\right)}\Gamma \left(m-1\right)$$

Substituting (71) into (65) gives the estimation using precautionary loss function as

(72) cˆPLF={ϑ2Γ(m)Γ(m−1)}12,m=2h+3n−12

#### 5.4.3 Estimation using Quadratic Loss Function (QLF)

For i=1, the numerator in (67) is

(73) $$E\left(c^{-1}﹨x\right)=\frac{\vartheta^{m}}{2^{m-1}\Gamma \left(m\right)}\int_{0}^{\infty }c^{-1-2h-3n}e^{-\frac{\vartheta }{2c^{2}}}dc$$

This can be written in (74) by applying (43) as

(74) $$E\left(c^{-1}﹨x\right)=\frac{\vartheta^{m-1}}{2^{m-1}\Gamma \left(m\right)}\int_{0}^{\infty }{\left({\left(\frac{\vartheta }{2\omega }\right)}^{\frac{1}{2}}\right)}^{2-2h-3n}e^{-\omega }d\omega$$

(75) $$=\frac{\vartheta^{m-1+\frac{2-2h-3n}{2}}}{2^{m-1+\frac{2-2h-3n}{2}}\Gamma \left(m\right)}\int_{0}^{\infty }\omega^{\frac{2h+3n-2}{2}}e^{-\omega }d\omega =\frac{\vartheta^{-\frac{1}{2}}}{2^{-\frac{1}{2}}\Gamma \left(m\right)}\Gamma \left(\frac{2h+3n}{2}\right)=\frac{\vartheta^{-\frac{1}{2}}}{2^{-\frac{1}{2}}\Gamma \left(m\right)}\Gamma \left(m+\frac{1}{2}\right)$$

When i=2, the denominator of equation (67) is

(76) $$E\left(c^{-2}﹨x\right)=\frac{\vartheta^{m}}{2^{m-1}\Gamma \left(m\right)}\int_{0}^{\infty }c^{-2-2h-3n}e^{-\frac{\vartheta }{2c^{2}}}dc$$

(77) $$=\frac{\vartheta^{m-1+\frac{1-2h-3n}{2}}}{2^{m-1+\frac{1-2h-3n}{2}}\Gamma \left(m\right)}\int_{0}^{\infty }\omega^{\left(\frac{2h+3n-1}{2}+1\right)-1}e^{-\omega }d\omega =\frac{\vartheta^{-1}}{2^{-1}\Gamma \left(m\right)}\Gamma \left(m+1\right)$$

Therefore, the Bayesian estimator of the scale parameter *c* using QLF is obtained by inserting (75) and (77) into (67) as

(78) $${\overset{ˆ}{c}}_{QLF}=\frac{\vartheta^{\frac{1}{2}}}{2^{\frac{1}{2}}}\frac{\Gamma \left(m+\frac{1}{2}\right)}{\Gamma \left(m+1\right)},\;\;\;\;\;\;\;\;\;\;\;\;\;\;\;\;\;\;m=\frac{2h+3n-1}{2}$$

### 5.5 Bayesian estimation of the scale parameter *c* of Maxwell-Mukherjee Islam distribution using assumption of Uniform prior under the three loss functions

Setting h=0, the estimate of the scale parameter *c* using the assumption of the uniform prior under the three loss functions considered in the study are given in this section.

#### 5.5.1 Estimation using Squared Error Loss Function (SELF)

As given in (69) for h=0, the estimate of the scale parameter *c* using the assumption of uniform prior under SELF is given by

(79) $${\overset{ˆ}{c}}_{SELF}=\frac{\Gamma \left(\frac{3n}{2}-1\right)}{\Gamma \left(\frac{3n}{2}-\frac{1}{2}\right)}\sqrt{\frac{\vartheta }{2}}$$

#### 5.5.2 Estimation using Precautionary Loss Function (PLF)

From (72), the estimate of the scale parameter *c* using the assumption of a uniform prior under PLF is given by

(80) $${\overset{ˆ}{c}}_{PLF}={\left\{\frac{\Gamma \left(\frac{3n}{2}-\frac{3}{2}\right)}{\Gamma \left(\frac{3n}{2}-\frac{1}{2}\right)}\right\}}^{\frac{1}{2}}\sqrt{\frac{\vartheta }{2}}$$

#### 5.5.3 Estimation using Quadratic Loss Function (QLF)

Equation (78) becomes the estimate of the scale parameter *c* using the assumption of a uniform prior under QLF as

(81) $${\overset{ˆ}{c}}_{QLF}=\frac{\Gamma \left(\frac{3n}{2}\right)}{\Gamma \left(\frac{3n}{2}+\frac{1}{2}\right)}\sqrt{\frac{\vartheta }{2}}$$

### 5.6 Bayesian estimation of the scale parameter *c* of Maxwell-Mukherjee Islam distribution using assumption of Jeffrey's prior under the three loss functions

The estimate of the scale parameter *c* using the assumption of Jeffrey's prior under the three loss functions given in this section by setting h=1/2.

#### 5.6.1 Estimation using Squared Error Loss Function (SELF)

As given in (69), the estimate of the scale parameter *c* using the assumption of Jeffrey's prior under SELF is given by

(82) $${\overset{ˆ}{c}}_{SELF}=\frac{\Gamma \left(\frac{3n}{2}-\frac{1}{2}\right)}{\Gamma \left(\frac{3n}{2}\right)}\sqrt{\frac{\vartheta }{2}}$$

#### 5.6.2 Estimation using Precautionary Loss Function (PLF)

From (72), the estimate of the scale parameter *c* using the assumption of Jeffrey's prior under PLF is given by

(83) $${\overset{ˆ}{c}}_{PLF}={\left\{\frac{\Gamma \left(\frac{3n}{2}-1\right)}{\Gamma \left(\frac{3n}{2}\right)}\right\}}^{\frac{1}{2}}\sqrt{\frac{\vartheta }{2}}$$

#### 5.6.3 Estimation using Quadratic Loss Function (QLF)

Equation (78) becomes the estimate of the scale parameter *c* using the assumption of Jeffrey's prior under QLF as

(84) $${\overset{ˆ}{c}}_{QLF}=\frac{\Gamma \left(\frac{3n}{2}+\frac{1}{2}\right)}{\Gamma \left(\frac{3n}{2}+1\right)}\sqrt{\frac{\vartheta }{2}}$$

### 5.7 Bayesian estimation of the scale parameter *c* of Maxwell-Mukherjee Islam distribution using assumption of Hartigan's prior under the three loss functions

The setting h=3/2, the estimate of the scale parameter *c* using the assumption of a Hartigan's prior under the three loss functions are given in this section.

#### 5.7.1 Using Squared Error Loss Function (SELF)

As given in (69), the estimate of the scale parameter *c* using the assumption of Hartigan's prior under SELF is given by

(85) $${\overset{ˆ}{c}}_{SELF}=\frac{\Gamma \left(\frac{3n}{2}+\frac{1}{2}\right)}{\Gamma \left(\frac{3n}{2}+1\right)}\sqrt{\frac{\vartheta }{2}}$$

#### 5.7.2 Using Precautionary Loss Function (PLF)

From (72), the estimate of the scale parameter *c* using the assumption of Hartigan's prior under PLF is given by

(86) $${\overset{ˆ}{c}}_{PLF}={\left\{\frac{\Gamma \left(\frac{3n}{2}\right)}{\Gamma \left(\frac{3n}{2}+1\right)}\right\}}^{\frac{1}{2}}\sqrt{\frac{\vartheta }{2}}$$

#### 5.7.3 Estimation using Quadratic Loss Function (QLF)

Equation (78) becomes the estimate of the scale parameter *c* using the assumption of Hartigan's prior under QLF as

(87) $${\overset{ˆ}{c}}_{QLF}=\frac{\Gamma \left(\frac{3n}{2}+\frac{3}{2}\right)}{\Gamma \left(\frac{3n}{2}+2\right)}\sqrt{\frac{\vartheta }{2}}$$

### 5.8 Bayesian estimation of the scale parameter *c* of Maxwell-Mukherjee Islam distribution using assumptions of the Inverse-Rayleigh prior under the three loss functions

#### 5.8.1 Estimation using Squared Error Loss Function (SELF)

By substituting posterior distribution defined in (55) into (63) gives

(88) $${\overset{ˆ}{c}}_{SELF}=\frac{2q^{f}}{\Gamma \left(f\right)}\int_{0}^{\infty }c^{1-3n-3}e^{-\frac{q}{c^{2}}}dc$$

Let

(89) $$s=\frac{q}{c^{2}},\;\;\;\;\;\;\;\;c={\left(\frac{q}{s}\right)}^{\frac{1}{2}}\;\;\;\;\;\;\;\;\Rightarrow \;\;\;\;\;\;\;\;dc=\frac{-c^{3}}{2q}ds$$

by inserting (89) into (88) becomes

(90) $${\overset{ˆ}{c}}_{SELF}=\frac{q^{f-1}}{\Gamma \left(f\right)}\int_{0}^{\infty }{\left({\left(\frac{q}{s}\right)}^{\frac{1}{2}}\right)}^{1-3n}e^{-s}ds=\frac{q^{f}}{q^{\frac{3n+1}{2}}\Gamma \left(f\right)}\int_{0}^{\infty }s^{\frac{3n-1}{2}}e^{-s}ds=\frac{q^{f}}{q^{\frac{3n+1}{2}}\Gamma \left(f\right)}\Gamma \left(\frac{3n+1}{2}\right)$$

#### 5.8.2 Estimation using Precautionary Loss Function (PLF)

The integral part of (65) is obtained in (91) by substituting posterior distribution defined in (55) as

(91) $$\int_{0}^{\infty }c^{2}\rho_{i}\left(c﹨X\right)dc=\frac{2q^{f}}{\Gamma \left(f\right)}\int_{0}^{\infty }c^{2-3n-3}e^{-\frac{q}{c^{2}}}dc$$

Using (89), (91) becomes

(92) $${\overset{ˆ}{c}}_{SELF}=\frac{q^{f-1}}{\Gamma \left(f\right)}\int_{0}^{\infty }{\left({\left(\frac{q}{s}\right)}^{\frac{1}{2}}\right)}^{2-3n}e^{-s}ds=\frac{q^{f}}{q^{\frac{3n}{2}}\Gamma \left(f\right)}\int_{0}^{\infty }s^{\frac{3n}{2}-1}e^{-s}ds=\frac{q^{f}}{q^{\frac{3n}{2}}\Gamma \left(f\right)}\Gamma \left(\frac{3n}{2}\right)$$

Substituting (92) into (65) gives the estimation using precautionary loss function as

(93) $${\overset{ˆ}{c}}_{PLF}={\left\{\frac{q^{f}}{q^{\frac{3n}{2}}\Gamma \left(f\right)}\Gamma \left(\frac{3n}{2}\right)\right\}}^{\frac{1}{2}}$$

#### 5.8.3 Estimation using Quadratic Loss Function (QLF)

For i=1, the numerator in (67) is

(94) $$E\left(c^{-1}﹨x\right)=\frac{2q^{f}}{\Gamma \left(f\right)}\int_{0}^{\infty }c^{-1-3n-3}e^{-\frac{q}{c^{2}}}dc$$

By applying (89), equation (94) can be expressed as

(95) $$E\left(c^{-1}﹨x\right)=\frac{q^{f-1}}{\Gamma \left(f\right)}\int_{0}^{\infty }{\left({\left(\frac{q}{s}\right)}^{\frac{1}{2}}\right)}^{-3n-1}e^{-s}ds=\frac{q^{f}}{q^{\frac{3n+3}{2}}\Gamma \left(f\right)}\int_{0}^{\infty }s^{\frac{3n+1}{2}}e^{-s}ds=\frac{q^{f}}{q^{\frac{3n+3}{2}}\Gamma \left(f\right)}\Gamma \left(\frac{3n+3}{2}\right)$$

When i=2, the denominator of equation (67) is

(96) $$E\left(c^{-2}﹨x\right)=\frac{q^{f-1}}{\Gamma \left(f\right)}\int_{0}^{\infty }{\left({\left(\frac{q}{s}\right)}^{\frac{1}{2}}\right)}^{-3n-2}e^{-s}ds=\frac{q^{f}}{q^{\frac{3n+4}{2}}\Gamma \left(f\right)}\int_{0}^{\infty }s^{\frac{3n+2}{2}}e^{-s}ds=\frac{q^{f}}{q^{\frac{3n+4}{2}}\Gamma \left(f\right)}\Gamma \left(\frac{3n+4}{2}\right)$$

Therefore, the Bayesian estimator of the scale parameter *c* using QLF is obtained by inserting (95) and (96) into (67) as

(97) $${\overset{ˆ}{c}}_{QLF}=\frac{q^{\frac{1}{2}}\Gamma \left(\frac{3n+3}{2}\right)}{\Gamma \left(\frac{3n+4}{2}\right)}$$

### 5.9 Bayesian estimation of the scale parameter *c* of Maxwell-Mukherjee Islam distribution using assumptions of the Inverse-Nakagami prior under the three loss functions

#### 5.9.1 Estimation using Squared Error Loss Function (SELF)

By substituting posterior distribution defined in (61) into (63) it becomes

(98) $${\overset{ˆ}{c}}_{SELF}=\frac{2t^{j}}{\Gamma \left(j\right)}\int_{0}^{\infty }c^{1-2\alpha -3n-1}e^{-\frac{t}{c^{2}}}dc$$

Let

(99) $$r=\frac{t}{c^{2}},\;\;\;\;\;\;\;\;c={\left(\frac{t}{r}\right)}^{\frac{1}{2}}\;\;\;\;\;\;\;\;\Rightarrow \;\;\;\;\;\;\;\;dc=\frac{-c^{3}}{2t}dr$$

by inserting (99) into (98) we get

(100) $${\overset{ˆ}{c}}_{SELF}=\frac{t^{j-1}}{\Gamma \left(j\right)}\int_{0}^{\infty }{\left({\left(\frac{t}{r}\right)}^{\frac{1}{2}}\right)}^{3-2\alpha -3n}e^{-r}dr=\frac{t^{j}}{t^{\frac{2\alpha +3n-1}{2}}\Gamma \left(j\right)}\int_{0}^{\infty }r^{\frac{2\alpha +3n-1}{2}-1}e^{-r}dr=\frac{t^{j}}{t^{\frac{2\alpha +3n-1}{2}}\Gamma \left(j\right)}\Gamma \left(\frac{2\alpha +3n-1}{2}\right)$$

#### 5.9.2 Estimation using Precautionary Loss Function (PLF)

The integral part of (65) is obtained in (101) by substituting posterior distribution defined in (61) as

(101) $$\int_{0}^{\infty }c^{2}\rho_{i}\left(c﹨X\right)dc=\frac{2t^{j}}{\Gamma \left(j\right)}\int_{0}^{\infty }c^{2-2\alpha -3n-1}e^{-\frac{t}{c^{2}}}dc$$

Using (99), equation (101) becomes

(102) $${\overset{ˆ}{c}}_{SELF}=\frac{t^{j-1}}{\Gamma \left(j\right)}\int_{0}^{\infty }{\left({\left(\frac{t}{r}\right)}^{\frac{1}{2}}\right)}^{4-2\alpha -3n}e^{-r}dr=\frac{t^{j}}{t^{\frac{2\alpha +3n-2}{2}}\Gamma \left(j\right)}\int_{0}^{\infty }r^{\frac{2\alpha +3n-2}{2}-1}e^{-r}dr=\frac{t^{j}}{t^{\frac{2\alpha +3n-2}{2}}\Gamma \left(j\right)}\Gamma \left(\frac{2\alpha +3n-2}{2}\right)$$

Substituting (102) into (65) gives the estimation using precautionary loss function as

(103) $${\overset{ˆ}{c}}_{PLF}={\left\{\frac{t^{j}}{t^{\frac{2\alpha +3n-2}{2}}\Gamma \left(j\right)}\Gamma \left(\frac{2\alpha +3n-2}{2}\right)\right\}}^{\frac{1}{2}}$$

#### 5.9.3 Estimation using Quadratic Loss Function (QLF)

For i=1, the numerator in (67) is

(104) $$E\left(c^{-1}﹨x\right)=\frac{2t^{j}}{\Gamma \left(j\right)}\int_{0}^{\infty }c^{-1-2\alpha -3n-1}e^{-\frac{t}{c^{2}}}dc$$

Equation (104) can be written in (105) by applying (99) as

(105) $$E\left(c^{-1}﹨x\right)=\frac{t^{j-1}}{\Gamma \left(j\right)}\int_{0}^{\infty }{\left({\left(\frac{t}{r}\right)}^{\frac{1}{2}}\right)}^{1-2\alpha -3n}e^{-r}dr$$

(106) $$=\frac{t^{j}}{t^{\frac{2\alpha +3n+1}{2}}\Gamma \left(j\right)}\int_{0}^{\infty }r^{\frac{2\alpha +3n+1}{2}-1}e^{-r}dr=\frac{t^{j}}{t^{\frac{2\alpha +3n+1}{2}}\Gamma \left(j\right)}\Gamma \left(\frac{2\alpha +3n+1}{2}\right)$$

When i=2, the denominator of (67) is

(107) $$E\left(c^{-2}﹨x\right)=\frac{t^{j-1}}{\Gamma \left(j\right)}\int_{0}^{\infty }{\left({\left(\frac{t}{r}\right)}^{\frac{1}{2}}\right)}^{-2\alpha -3n}e^{-r}dr=\frac{t^{j}}{t^{\frac{2\alpha +3n+2}{2}}\Gamma \left(j\right)}\int_{0}^{\infty }r^{\frac{2\alpha +3n}{2}}e^{-r}dr=\frac{t^{j}}{t^{\frac{2\alpha +3n+2}{2}}\Gamma \left(j\right)}\Gamma \left(\frac{2\alpha +3n+2}{2}\right)$$

Therefore, the Bayesian estimator of the scale parameter *c* using QLF is obtained by inserting (106) and (107) into (67) as

(108) $${\overset{ˆ}{c}}_{QLF}=\frac{t^{\frac{1}{2}}\Gamma \left(\frac{2\alpha +3n+1}{2}\right)}{\Gamma \left(\frac{2\alpha +3n+2}{2}\right)}$$

The summary of the mathematical derivatives of the SELF, PLF and QLF under assumptions of Uniform, Jeffrey's, Hartigan's, Inverse-Rayleigh and Inverse-Nakagami priors are provided in Table 1.

**Table 1:** Summary of the mathematical expressions for the assumptions of different priors and loss functions.

Priors	Loss Function		
	SELF	PLF	QLF
Uniform	$\frac{\Gamma \left(\frac{3n}{2}-1\right)}{\Gamma \left(\frac{3n}{2}-\frac{1}{2}\right)}\sqrt{\frac{\vartheta }{2}}$	${\left\{\frac{\Gamma \left(\frac{3n}{2}-\frac{3}{2}\right)}{\Gamma \left(\frac{3n}{2}-\frac{1}{2}\right)}\right\}}^{\frac{1}{2}}\sqrt{\frac{\vartheta }{2}}$	$\frac{\Gamma \left(\frac{3n}{2}\right)}{\Gamma \left(\frac{3n}{2}+\frac{1}{2}\right)}\sqrt{\frac{\vartheta }{2}}$
Jeffrey's	$\frac{\Gamma \left(\frac{3n}{2}-\frac{1}{2}\right)}{\Gamma \left(\frac{3n}{2}\right)}\sqrt{\frac{\vartheta }{2}}$	${\left\{\frac{\Gamma \left(\frac{3n}{2}-1\right)}{\Gamma \left(\frac{3n}{2}\right)}\right\}}^{\frac{1}{2}}\sqrt{\frac{\vartheta }{2}}$	$\frac{\Gamma \left(\frac{3n}{2}+\frac{1}{2}\right)}{\Gamma \left(\frac{3n}{2}+1\right)}\sqrt{\frac{\vartheta }{2}}$
Hartigan's	$\frac{\Gamma \left(\frac{3n}{2}+\frac{1}{2}\right)}{\Gamma \left(\frac{3n}{2}+1\right)}\sqrt{\frac{\vartheta }{2}}$	${\left\{\frac{\Gamma \left(\frac{3n}{2}\right)}{\Gamma \left(\frac{3n}{2}+1\right)}\right\}}^{\frac{1}{2}}\sqrt{\frac{\vartheta }{2}}$	$\frac{\Gamma \left(\frac{3n}{2}+\frac{3}{2}\right)}{\Gamma \left(\frac{3n}{2}+2\right)}\sqrt{\frac{\vartheta }{2}}$
Inverse-Rayleigh	$\frac{q^{f}\Gamma \left(\frac{3n+1}{2}\right)}{q^{\frac{3n+1}{2}}\Gamma \left(f\right)}$	${\left\{\frac{q^{f}\Gamma \left(\frac{3n}{2}\right)}{q^{\frac{3n}{2}}\Gamma \left(f\right)}\right\}}^{\frac{1}{2}}$	$\frac{q^{\frac{1}{2}}\Gamma \left(\frac{3n+3}{2}\right)}{\Gamma \left(\frac{3n+4}{2}\right)}$
Inverse-Nakagami	$\frac{t^{j}\Gamma \left(\frac{2\alpha +3n-1}{2}\right)}{t^{\frac{2\alpha +3n-1}{2}}\Gamma \left(j\right)}$	${\left\{\frac{t^{j}\Gamma \left(\frac{2\alpha +3n-2}{2}\right)}{t^{\frac{2\alpha +3n-2}{2}}\Gamma \left(j\right)}\right\}}^{\frac{1}{2}}$	$\frac{t^{\frac{1}{2}}\Gamma \left(\frac{2\alpha +3n+1}{2}\right)}{\Gamma \left(\frac{2\alpha +3n+2}{2}\right)}$

Where $j=\frac{2\alpha +3n}{2}$, $t=\frac{\alpha }{\beta }+\frac{\vartheta }{2}$, $f=1+\frac{3n}{2}$ and $q=\lambda +\frac{\vartheta }{2}$.

As observed in Table 1, the mathematical derivatives of the QLF and SELF under Jeffrey's and Hartigan's priors produced the same estimates.

## 6 Simulation study and application to data set

This section provides quantile function and a simulation study of the proposed Maxwell-Mukherjee Islam distribution.

### 6.1 Quantile function

The quantile function of the Maxwell-Mukherjee Islam distribution is obtained by inverting the cdf defined in (5) as

(109) $$x_{q}=a{\left(\frac{{\left[2c^{2}\gamma^{-1}\left(\frac{3}{2},u\Gamma \left(\frac{3}{2}\right)\right)\right]}^{\frac{1}{2}}}{1+{\left[2c^{2}\gamma^{-1}\left(\frac{3}{2},u\Gamma \left(\frac{3}{2}\right)\right)\right]}^{\frac{1}{2}}}\right)}^{\frac{1}{b}}$$

where *u* has a uniform random variable defined on the interval 0 to 1.

### 6.2 Simulation study

A simulation study was conducted based on quantile function defined in (109) at sample sizes 10, 15, 20, 30, and 50, and for parameter values a=c=α=β=λ=1 and b=1.5. The simulation study was repeated 10000 times in which the estimate of scale parameter *c*, bias and mean squared errors (MSE) using assumptions of Uniform, Jeffrey's, Hartigan's, I-R and I-N priors under the three loss functions are obtained. Table 2 presents the estimate, bias, and MSE using assumptions of the different priors under the three loss functions for various parameter values.

**Table 2:** The Estimate, bias and MSE using different priors and loss functions at *a* = *c* = *λ* = *α* = *β* = 1 and *b* = 1.5.

n	Measure	Uniform			Jeffre'y			Hartigan's			I-R			I-N		
		SELF	PLF	QLF	SELF	PLF	QLF	SELF	PLF	QLF	SELF	PLF	QLF	SELF	PLF	QLF
10	Estimate	1.0364	1.0460	1.0006	1.0180	1.0272	0.9841	0.9841	0.9923	0.9533	1.0174	1.0259	0.9856	1.0174	1.0259	0.9856
	BIAS	-0.0364	-0.0460	-0.0006	-0.0180	-0.0272	0.0159	0.0159	0.0077	0.0467	-0.0174	-0.0259	0.0144	-0.0174	-0.0259	0.0144
	MSE	0.0192	0.0204	0.0167	0.0176	0.0183	0.0164	0.0164	0.0165	0.0173	0.0154	0.0160	0.0144	0.0154	0.0160	0.0144
15	Estimate	1.0236	1.0297	1.0004	1.0118	1.0177	0.9893	0.9893	0.9948	0.9683	1.0115	1.0172	0.9900	1.0115	1.0172	0.9900
	BIAS	-0.0236	-0.0297	-0.0004	-0.0118	-0.0177	0.0107	0.0107	0.0052	0.0317	-0.0115	-0.0172	0.0100	-0.0115	-0.0172	0.0100
	MSE	0.0125	0.0130	0.0114	0.0118	0.0121	0.0113	0.0113	0.0113	0.0117	0.0108	0.0111	0.0103	0.0108	0.0111	0.0103
20	Estimate	1.0176	1.0221	1.0003	1.0089	1.0132	0.9920	0.9920	0.9962	0.9760	1.0087	1.0129	0.9924	1.0087	1.0129	0.9924
	BIAS	-0.0176	-0.0221	-0.0003	-0.0089	-0.0132	0.0080	0.0080	0.0038	0.0240	-0.0087	-0.0129	0.0076	-0.0087	-0.0129	0.0076
	MSE	0.0090	0.0093	0.0084	0.0086	0.0088	0.0083	0.0083	0.0083	0.0086	0.0081	0.0082	0.0078	0.0081	0.0082	0.0078
30	Estimate	1.0111	1.0141	0.9998	1.0054	1.0083	0.9942	0.9942	0.9970	0.9834	1.0054	1.0082	0.9944	1.0054	1.0082	0.9944
	BIAS	-0.0111	-0.0141	0.0002	-0.0054	-0.0083	0.0058	0.0058	0.0030	0.0166	-0.0054	-0.0082	0.0056	-0.0054	-0.0082	0.0056
	MSE	0.0057	0.0058	0.0055	0.0056	0.0057	0.0055	0.0055	0.0055	0.0056	0.0053	0.0054	0.0052	0.0053	0.0054	0.0052
50	Estimate	1.0065	1.0082	0.9998	1.0031	1.0048	0.9965	0.9965	0.9981	0.9899	1.0031	1.0048	0.9965	1.0031	1.0048	0.9965
	BIAS	-0.0065	-0.0082	0.0002	-0.0031	-0.0048	0.0035	0.0035	0.0019	0.0101	-0.0031	-0.0048	0.0035	-0.0031	-0.0048	0.0035
	MSE	0.0034	0.0034	0.0033	0.0033	0.0033	0.0033	0.0033	0.0033	0.0033	0.0032	0.0033	0.0032	0.0032	0.0033	0.0032

As we observe from Table 2, QLF and SELF under Jeffrey's and Hartigan's priors produced the same estimate, bias, and MSE irrespective of the sample sizes. Similarly, SELF, PLF and QLF under I-R and I-N priors produced similar results. The table shows that QLF under I-R and I-N priors gave the minimum MSE, and then followed by SELF and PLF under I-R and I-N priors. As sample size increased, the MSE value of each method under Uniform, Jeffrey's, Hartigan's, I-R and I-N priors decreased and approached similar values.

The estimate, bias, and MSE using assumptions of Uniform, Jeffrey's, Hartigan's, I-R and I-N priors under the three loss functions for parameter values a=c=λ=β=1, b=1.5 and α=2 are presented in Table 3.

**Table 3:** The Estimate, bias and MSE using different priors and loss functions at *a* = *c* = *λ* = *β* = 1, *b* = 1.5 and *α* = 2.

n	Measure	Uniform			Jeffre'y			Hartigan's			I-R			I-N		
		SELF	PLF	QLF	SELF	PLF	QLF	SELF	PLF	QLF	SELF	PLF	QLF	SELF	PLF	QLF
10	Estimate	1.0364	1.0460	1.0006	1.0180	1.0272	0.9841	0.9841	0.9923	0.9533	1.0174	1.0259	0.9856	1.0167	1.0247	0.9868
	BIAS	-0.0364	-0.0460	-0.0006	-0.0180	-0.0272	0.0159	0.0159	0.0077	0.0467	-0.0174	-0.0259	0.0144	-0.0167	-0.0247	0.0132
	MSE	0.0192	0.0204	0.0167	0.0176	0.0183	0.0164	0.0164	0.0165	0.0173	0.0154	0.0160	0.0144	0.0136	0.0141	0.0127
15	Estimate	1.0236	1.0297	1.0004	1.0118	1.0177	0.9893	0.9893	0.9948	0.9683	1.0115	1.0172	0.9900	1.0113	1.0166	0.9906
	BIAS	-0.0236	-0.0297	-0.0004	-0.0118	-0.0177	0.0107	0.0107	0.0052	0.0317	-0.0115	-0.0172	0.0100	-0.0113	-0.0166	0.0094
	MSE	0.0125	0.0130	0.0114	0.0118	0.0121	0.0113	0.0113	0.0113	0.0117	0.0108	0.0111	0.0103	0.0099	0.0102	0.0095
20	Estimate	1.0176	1.0221	1.0003	1.0089	1.0132	0.9920	0.9920	0.9962	0.9760	1.0087	1.0129	0.9924	1.0086	1.0126	0.9928
	BIAS	-0.0176	-0.0221	-0.0003	-0.0089	-0.0132	0.0080	0.0080	0.0038	0.0240	-0.0087	-0.0129	0.0076	-0.0086	-0.0126	0.0072
	MSE	0.0090	0.0093	0.0084	0.0086	0.0088	0.0083	0.0083	0.0083	0.0086	0.0081	0.0082	0.0078	0.0076	0.0077	0.0073
30	Estimate	1.0111	1.0141	0.9998	1.0054	1.0083	0.9942	0.9942	0.9970	0.9834	1.0054	1.0082	0.9944	1.0053	1.0080	0.9946
	BIAS	-0.0111	-0.0141	0.0002	-0.0054	-0.0083	0.0058	0.0058	0.0030	0.0166	-0.0054	-0.0082	0.0056	-0.0053	-0.0080	0.0054
	MSE	0.0057	0.0058	0.0055	0.0056	0.0057	0.0055	0.0055	0.0055	0.0056	0.0053	0.0054	0.0052	0.0051	0.0052	0.0050
50	Estimate	1.0065	1.0082	0.9998	1.0031	1.0048	0.9965	0.9965	0.9981	0.9899	1.0031	1.0048	0.9965	1.0031	1.0048	0.9966
	BIAS	-0.0065	-0.0082	0.0002	-0.0031	-0.0048	0.0035	0.0035	0.0019	0.0101	-0.0031	-0.0048	0.0035	-0.0031	-0.0048	0.0034
	MSE	0.0034	0.0034	0.0033	0.0033	0.0033	0.0033	0.0033	0.0033	0.0033	0.0032	0.0033	0.0032	0.0031	0.0032	0.0031

We notice from Table 3 that QLF and SELF under Jeffrey's and Hartigan's priors produced similar pattern just like Table 2. In this case, the QLF under I-N prior gave the minimum value of MSE irrespective of the sample size n. However, as the sample size increased, the MSE value of each method under the different priors decreased and approached similar values.

Keeping a=c=λ=α=1, b=1.5 and β=1.5, the estimate, bias, and MSE using assumptions of different priors under loss functions are presented in Table 4.

**Table 4:** The Estimate, bias and MSE using different priors and loss functions at *a* = *c* = *λ* = *α* = 1, *b* = 1.5 and *β* = 1.5.

n	Measure	Uniform			Jeffre'y			Hartigan's			I-R			I-N		
		SELF	PLF	QLF	SELF	PLF	QLF	SELF	PLF	QLF	SELF	PLF	QLF	SELF	PLF	QLF
10	Estimate	1.0364	1.0460	1.0006	1.0180	1.0272	0.9841	0.9841	0.9923	0.9533	1.0174	1.0259	0.9856	1.0064	1.0148	0.9750
	BIAS	-0.0364	-0.0460	-0.0006	-0.0180	-0.0272	0.0159	0.0159	0.0077	0.0467	-0.0174	-0.0259	0.0144	-0.0064	-0.0148	0.0250
	MSE	0.0192	0.0204	0.0167	0.0176	0.0183	0.0164	0.0164	0.0165	0.0173	0.0154	0.0160	0.0144	0.0155	0.0159	0.0151
15	Estimate	1.0236	1.0297	1.0004	1.0118	1.0177	0.9893	0.9893	0.9948	0.9683	1.0115	1.0172	0.9900	1.0042	1.0098	0.9828
	BIAS	-0.0236	-0.0297	-0.0004	-0.0118	-0.0177	0.0107	0.0107	0.0052	0.0317	-0.0115	-0.0172	0.0100	-0.0042	-0.0098	0.0172
	MSE	0.0125	0.0130	0.0114	0.0118	0.0121	0.0113	0.0113	0.0113	0.0117	0.0108	0.0111	0.0103	0.0108	0.0110	0.0106
20	Estimate	1.0176	1.0221	1.0003	1.0089	1.0132	0.9920	0.9920	0.9962	0.9760	1.0087	1.0129	0.9924	1.0032	1.0074	0.987
	BIAS	-0.0176	-0.0221	-0.0003	-0.0089	-0.0132	0.0080	0.0080	0.0038	0.0240	-0.0087	-0.0129	0.0076	-0.0032	-0.0074	0.013
	MSE	0.0090	0.0093	0.0084	0.0086	0.0088	0.0083	0.0083	0.0083	0.0086	0.0081	0.0082	0.0078	0.0081	0.0082	0.008
30	Estimate	1.0111	1.0141	0.9998	1.0054	1.0083	0.9942	0.9942	0.9970	0.9834	1.0054	1.0082	0.9944	1.0017	1.0045	0.9908
	BIAS	-0.0111	-0.0141	0.0002	-0.0054	-0.0083	0.0058	0.0058	0.0030	0.0166	-0.0054	-0.0082	0.0056	-0.0017	-0.0045	0.0092
	MSE	0.0057	0.0058	0.0055	0.0056	0.0057	0.0055	0.0055	0.0055	0.0056	0.0053	0.0054	0.0052	0.0054	0.0054	0.0053
50	Estimate	1.0065	1.0082	0.9998	1.0031	1.0048	0.9965	0.9965	0.9981	0.9899	1.0031	1.0048	0.9965	1.0009	1.0026	0.9943
	BIAS	-0.0065	-0.0082	0.0002	-0.0031	-0.0048	0.0035	0.0035	0.0019	0.0101	-0.0031	-0.0048	0.0035	-0.0009	-0.0026	0.0057
	MSE	0.0034	0.0034	0.0033	0.0033	0.0033	0.0033	0.0033	0.0033	0.0033	0.0032	0.0033	0.0032	0.0032	0.0033	0.0032

In Table 4, the results of QLF and SELF under Jeffrey's and Hartigan's priors followed similar pattern as we found in Table 2, Table 3. It is observed that QLF under Inverse-Rayleigh prior had minimum value of MSE followed by QLF under I-N, SELF under I-R. As the sample size increased, the MSE of each method under different priors approached the same results.

### 6.3 Application to real life data set

This section provides an application to real life data set.

The real life data set comprises 22 observations of the end yearly selling Nigerian Naira to Japanese Yen exchange rates from 1995 to 2016. The data were recently studied by [16] and [17] and are presented as follows:

0.8308, 0.7032, 0.5755, 0.6493, 0.7597, 0.9546, 0.8639, 1.0694, 1.2818, 1.2969, 1.0982, 1.0672, 1.0412, 1.4506, 1.6025, 1.6988, 2.0259, 1.8803, 1.5759, 1.4676, 1.6307, 2.5317.

Using this data, a dataset was generated from Maxwell-Mukherjee Islam distribution for different parameter values β=1, 1.5, 2, 2.5 and 3. The estimate, bias, and MSE using assumptions of the Uniform, Jeffre's, Hartigan's, Inverse-Rayleyh and Inverse-Nakagami priors under the three loss functions were computed by setting a=c=λ=α=1 and b=1.5, and the results are presented in 5.

**Table 5:** The Estimate, bias and MSE using different priors and loss functions at *a* = *c* = *λ* = *α* = 1 and *b* = 1.5.

*β*	Measure	Uniform			Jeffre'y			Hartigan's			I-R			I-N		
		SELF	PLF	QLF	SELF	PLF	QLF	SELF	PLF	QLF	SELF	PLF	QLF	SELF	PLF	QLF
1	Estimate	1.0162	1.0202	1.0005	1.0083	1.0122	0.9930	0.9930	0.9968	0.9784	1.0081	1.0120	0.9933	1.0081	1.0120	0.9933
	BIAS	-0.0162	-0.0202	-0.0005	-0.0083	-0.0122	0.0070	0.0070	0.0032	0.0216	-0.0081	-0.0120	0.0067	-0.0081	-0.0120	0.0067
	MSE	0.0081	0.0083	0.0076	0.0078	0.0080	0.0076	0.0076	0.0076	0.0078	0.0074	0.0075	0.0071	0.0074	0.0075	0.0071
1.5	Estimate	1.0164	1.0205	1.0008	1.0085	1.0125	0.9932	0.9932	0.9970	0.9786	1.0084	1.0122	0.9936	1.0034	1.0072	0.9886
	BIAS	-0.0164	-0.0205	-0.0008	-0.0085	-0.0125	0.0068	0.0068	0.0030	0.0214	-0.0084	-0.0122	0.0064	-0.0034	-0.0072	0.0114
	MSE	0.0082	0.0084	0.0077	0.0078	0.0080	0.0076	0.0076	0.0076	0.0078	0.0074	0.0075	0.0071	0.0074	0.0075	0.0073
2	Estimate	1.0165	1.0205	1.0008	1.0086	1.0125	0.9933	0.9933	0.9970	0.9787	1.0084	1.0122	0.9936	1.0009	1.0047	0.9861
	BIAS	-0.0165	-0.0205	-0.0008	-0.0086	-0.0125	0.0067	0.0067	0.0030	0.0213	-0.0084	-0.0122	0.0064	-0.0009	-0.0047	0.0139
	MSE	0.0082	0.0084	0.0077	0.0079	0.0080	0.0076	0.0076	0.0077	0.0078	0.0074	0.0076	0.0072	0.0075	0.0076	0.0075
2.5	Estimate	1.0158	1.0198	1.0001	1.0079	1.0118	0.9926	0.9926	0.9963	0.9780	1.0077	1.0116	0.9929	0.9987	1.0025	0.9840
	BIAS	-0.0158	-0.0198	-0.0001	-0.0079	-0.0118	0.0074	0.0074	0.0037	0.0220	-0.0077	-0.0116	0.0071	0.0013	-0.0025	0.0160
	MSE	0.0079	0.0081	0.0074	0.0076	0.0077	0.0074	0.0074	0.0074	0.0076	0.0071	0.0073	0.0069	0.0072	0.0073	0.0073
3	Estimate	1.0159	1.0199	1.0003	1.0080	1.0120	0.9927	0.9927	0.9965	0.9781	1.0079	1.0117	0.9931	0.9978	1.0016	0.9831
	BIAS	-0.0159	-0.0199	-0.0003	-0.0080	-0.0120	0.0073	0.0073	0.0035	0.0219	-0.0079	-0.0117	0.0069	0.0022	-0.0016	0.0169
	MSE	0.0079	0.0081	0.0074	0.0076	0.0077	0.0073	0.0073	0.0073	0.0075	0.0071	0.0072	0.0069	0.0072	0.0073	0.0073

As observed from 5, both QLF and SELF under Jeffrey's and Hartigan's priors produced the same estimate, bias, and MSE irrespective of different parameter values. Similarly, the SELF, PLF and QLF under I-R and I-N priors performed similarly when the parameter β=1. As parameter *β* increased, the MSE value of each method under the different priors decreased and approached similar values. Also, the table shows that the QLF under I-R prior gave the minimum value of MSE.

Keeping a=c=λ=β=1 and b=1.5, the estimate, bias and MSE using assumptions of the Uniform, Jeffrey's, Hartigan's, I-R and I-N priors are provided in Table 6

**Table 6:** The Estimate, bias and MSE using different priors and loss functions at *a* = *c* = *λ* = *β* = 1 and *b* = 1.5.

*α*	Measure	Uniform			Jeffre'y			Hartigan's			I-R			I-N		
		SELF	PLF	QLF	SELF	PLF	QLF	SELF	PLF	QLF	SELF	PLF	QLF	SELF	PLF	QLF
1	Estimate	1.0162	1.0202	1.0005	1.0083	1.0122	0.9930	0.9930	0.9968	0.9784	1.0081	1.0120	0.9933	1.0081	1.0120	0.9933
	BIAS	-0.0162	-0.0202	-0.0005	-0.0083	-0.0122	0.0070	0.0070	0.0032	0.0216	-0.0081	-0.0120	0.0067	-0.0081	-0.0120	0.0067
	MSE	0.0081	0.0083	0.0076	0.0078	0.0080	0.0076	0.0076	0.0076	0.0078	0.0074	0.0075	0.0071	0.0074	0.0075	0.0071
1.5	Estimate	1.0164	1.0205	1.0008	1.0085	1.0125	0.9932	0.9932	0.9970	0.9786	1.0084	1.0122	0.9936	1.0083	1.0121	0.9937
	BIAS	-0.0164	-0.0205	-0.0008	-0.0085	-0.0125	0.0068	0.0068	0.0030	0.0214	-0.0084	-0.0122	0.0064	-0.0083	-0.0121	0.0063
	MSE	0.0082	0.0084	0.0077	0.0078	0.0080	0.0076	0.0076	0.0076	0.0078	0.0074	0.0075	0.0071	0.0072	0.0073	0.0069
2	Estimate	1.0165	1.0205	1.0008	1.0086	1.0125	0.9933	0.9933	0.9970	0.9787	1.0084	1.0122	0.9936	1.0083	1.0120	0.9939
	BIAS	-0.0165	-0.0205	-0.0008	-0.0086	-0.0125	0.0067	0.0067	0.0030	0.0213	-0.0084	-0.0122	0.0064	-0.0083	-0.0120	0.0061
	MSE	0.0082	0.0084	0.0077	0.0079	0.0080	0.0076	0.0076	0.0077	0.0078	0.0074	0.0076	0.0072	0.0070	0.0071	0.0068
2.5	Estimate	1.0158	1.0198	1.0001	1.0079	1.0118	0.9926	0.9926	0.9963	0.9780	1.0077	1.0116	0.9929	1.0075	1.0112	0.9933
	BIAS	-0.0158	-0.0198	-0.0001	-0.0079	-0.0118	0.0074	0.0074	0.0037	0.0220	-0.0077	-0.0116	0.0071	-0.0075	-0.0112	0.0067
	MSE	0.0079	0.0081	0.0074	0.0076	0.0077	0.0074	0.0074	0.0074	0.0076	0.0071	0.0073	0.0069	0.0065	0.0067	0.0064
3	Estimate	1.0159	1.0199	1.0003	1.0080	1.0120	0.9927	0.9927	0.9965	0.9781	1.0079	1.0117	0.9931	1.0076	1.0112	0.9936
	BIAS	-0.0159	-0.0199	-0.0003	-0.0080	-0.0120	0.0073	0.0073	0.0035	0.0219	-0.0079	-0.0117	0.0069	-0.0076	-0.0112	0.0064
	MSE	0.0079	0.0081	0.0074	0.0076	0.0077	0.0073	0.0073	0.0073	0.0075	0.0071	0.0072	0.0069	0.0063	0.0064	0.0061

It is observed from Table 6 that the QLF and SELF under Jeffrey's and Hartigan's priors produced similar results irrespective of different parameter values and also, the SELF, PLF and QLF under I-R and I-N priors produced similar results when the parameter α=1. By increasing the value of *α*, the MSE value of each method under the different priors decreased and approached similar values.

## 7 Discussion of results and conclusion

This article proposed and studied Maxwell-Mukherjee Islam distribution based on the family of Maxwell generalized class of distributions. Bayesian estimation of the scale parameter *c* of the Maxwell-Mukherjee Islam distribution was carried out under Uniform, Jeffrey's, Hartigan's, Inverse-Rayleigh and Inverse-Nakagami priors using three loss functions, namely; squared error, quadratic and precautionary loss functions. As observed from the simulation results, the SELF, PLF and QLF under Inverse-Rayleigh and Inverse-Nakagami priors produced similar results when some parameters are the same. For different sample sizes and parameter values, QLF under Inverse-Rayleigh and Inverse-Nakagami priors produced smaller MSEs compared to other priors. In the application to real life data set, the QLF and SELF under Jeffrey's and Hartigan's priors produced the same estimate, bias and MSE irrespective of different parameter values. Similarly, the SELF, PLF and QLF under Inverse-Rayleigh and Inverse-Nakagami priors also provided similar results for some parameter values. It can therefore be concluded that the QLF under Inverse-Rayleigh and Inverse-Nakagami priors could be employed effectively in estimating the scale parameter *c* of the Maxwell-Mukherjee Islam distribution.

## Declarations

### Author contribution statement

Aliyu Ismail Ishaq, Alfred Adewole Abiodun, Jamilu Yunusa Falgore: Conceived and designed the experiments; Performed the experiments; Analyzed and interpreted the data; Contributed reagents, materials, analysis tools or data; Wrote the paper.

### Funding statement

This research did not receive any specific grant from funding agencies in the public, commercial, or not-for-profit sectors.

### Data availability statement

Data included in article/supplementary material/referenced in article.

### Declaration of interests statement

The authors declare no conflict of interest.

### Additional information

No additional information is available for this paper.
